# Assessing the acceptability of COVID‐19 vaccine and its booster dose

**DOI:** 10.1002/iid3.950

**Published:** 2023-09-26

**Authors:** Sawsan Mubarak, Ashraf A'aqoulah, Hadeel AlGhawrie, Samir Albalas, Nisreen Innab

**Affiliations:** ^1^ Infection Control Program King Hussein Cancer Center Amman Jordan; ^2^ Department of Health Systems Management, College of Public Health and Health Informatics King Saud bin Abdulaziz University for Health Sciences Riyadh Saudi Arabia; ^3^ King Abdullah International Medical Research Center Riyadh Saudi Arabia; ^4^ Department of Health Services Administration, Faculty of Economics and Administrative Sciences Yarmouk University Irbid Jordan; ^5^ Department of Computer Science and Information Systems, College of Applied Sciences AlMaarefa University Saudi Arabia

**Keywords:** booster dose, coronavirus, COVID‐19, healthcare, immunity, pandemic, vaccine

## Abstract

**Background and Objective:**

Coronavirus disease of 2019 (COVID‐19) vaccinations are essential to control the pandemic and prevent severe COVID‐19 infections. This study aims to assess the acceptability of the COVID‐19 vaccine and the factors that impact the intention to take the COVID‐19 vaccine and its booster dose.

**Methods:**

A cross‐sectional study was conducted in Saudi Arabia and Jordan. The study used a self‐administered web‐based survey (questionnaire) for data collection that was distributed via social media platforms from May 2022 to July 2022.

**Results:**

In this study, among 518 participants, 54.4% had already received two doses of the COVID‐19 vaccine, and out of the participants who didn't receive the booster dose, 19.9% declared a definite willingness to receive it, while 42% had already taken a booster dose, which indicated good acceptance. After adjustment for significant background characteristics, a significant association between the country and receiving the COVID‐19 vaccine, the intention to get the vaccine, and infection with COVID‐19 were found, in addition to a significant association between the country and the participants' opinion that electronic applications helped them to follow their vaccine schedule were found (*p* < .001). Also, the results showed that participants' attitudes were significantly associated with educational level and age groups (*p* ≤ .001, *p* = .032, respectively). There was a significant association between the intention to receive the vaccine booster dose and the country (*p* < .001). The Saudi participants were willing to get the booster dose seven times more than the Jordanians, furthermore, there was a significant association between taking the vaccine booster dose in the country, as well as age group, working in the medical field, previous COVID‐19 infection, and the intention to vaccinate the children (*p* < .001, *p* = .030, .031, .025, < .001, respectively).

**Conclusion:**

Overall, our results emphasize a positive response and a positive attitude toward COVID‐19 vaccination. In addition, define the groups to be targeted with effective communication regarding the COVID‐19 vaccine and its booster dose.

## 1 INTRODUCTION

The coronavirus disease of 2019 (COVID‐19) appeared on December 12, 2019, in the Chinese city of Wuhan.^1^ The virus has spread rapidly to adjacent countries and even farther afield, leading the World Health Organization (WHO) to declare the epidemic as a pandemic on March 11, 2020, 3 months after it was initially identified as an outbreak.^2^, ^3^ The devastating pandemic caused by severe acute respiratory syndrome coronavirus 2 (SARS‐CoV‐2) has been associated with large numbers of cases, mortality, and severe long‐term complications influencing individuals and economies.^3^ By early 2021, COVID‐19 vaccines had been produced and exhibited excellent protection against COVID‐19 infection, hospitalizations, and deaths.^4^ First, only four COVID‐19 vaccines were authorized in all countries of the world: BNT162 (Pfizer Biontech), mRNA1273 (Moderna), ChAdOx1 (AstraZeneca), and Ad26. COV2‐S (Johnson & Johnson), Other vaccines such as Sputnik V (Gamaleya), BBIBP‐CorV (Sinopharm), CoronaVac (Sinovac), and COVAXIN (Bharat Biotech) have also finished phase III clinical trials and are currently authorized for emergency use in many countries.^5^

The rapid development of COVID‐19 vaccines, due to the urgency of the pandemic, in addition to the technological advances used in the vaccines, has contributed to COVID‐19 vaccine hesitancy.^6^ However, clinical research and real‐world evidence suggest that vaccines are highly effective at preventing symptomatic disease in a few months.^7^ There has been considerable discussion about the eventual need for a booster dose of the COVID‐19 vaccine, primarily in response to concerns about the possible introduction of novel virus variations with greater transmissibility and diminishing immunity, in which several studies appeared to show a decline in protection against COVID‐19 infection a few months after the initial vaccination.^8^, ^9^, ^10^ These mutations were associated with increased recurrence of infection, transmissibility, and immune evasion even after natural immunity caused by infection or vaccine‐induced immunity. Booster doses were shown to provide a fast and significant improvement in protection against infection for all variants as they improved humoral and cellular immunity against different variants.^1^

A study showed that with the emergence of new SARS‐CoV‐2 variants, dominated by the highly transmissible Delta (B.1.617.2) variant, the efficacy of vaccines against infection with this variant and other so‐called “variants of concern” was found to be reduced,^11^ revealing the need for a third dose that is active against new variants. Many countries have already started administering booster doses in the wake of breakthrough infections, the arrival of new variants, and a decline in long‐term protection. Booster dosages will play an important part in the public health response to the pandemic at some point. The public's acceptance of a booster dose, on the other hand, appears to be a potential worry. Hesitancy and mistrust have impeded COVID‐19 immunization attempts.^12^, ^13^ Vaccine hesitancy is defined by the Strategic Advisory Group of Experts on Immunization (SAGE) as the delay in acceptance or refusal of vaccination despite the availability of vaccination services. Vaccine hesitancy is complex and context‐specific, varying across time, place, and vaccines. It is influenced by factors such as complacency, convenience, and confidence.^1^

Previous studies demonstrated the escalating worldwide occurrence of vaccine hesitancy in general as a global phenomenon.^14^, ^15^ Prominent factors behind vaccine hesitancy identified include safety issues, religious beliefs, beliefs that vaccines do not work, and overall lack of information.^16^

A study based on a sample from 19 countries showed that the global acceptance of COVID‐19 vaccines ranges between as low as 54.8% from Russia to as high as 88.6% from China. Moreover, most western countries report relatively higher public acceptance (59%–75%).^17^

A study in Hong Kong found that about half of older adults were hesitant to receive the second COVID‐19 vaccine booster dose. The background characteristics, such as perceived benefits, cues to action, and perceived self‐efficacy of receiving the second booster dose, were associated with lower vaccine hesitancy. Perceived barriers to fatigue from receiving repeated COVID‐19 vaccinations were associated with higher vaccine hesitancy.^18^ Another study in Hong Kong showed that 31.6% of older adult participants received a COVID‐19 vaccine booster dose.^19^ In a study conducted in the United States, the acceptance of the booster dose among healthcare workers was 83.6% when asked about acceptance of a hypothetical yearly booster vaccine to maintain immunity.^20^ In Poland, 71% of adults expressed a willingness to accept the booster dose, which was increased particularly in those having a worse experience with past COVID‐19 vaccination.^21^ In Japan, the booster vaccine for COVID‐19 was willingly received by 84.5% of Japanese medical students.^22^

A study conducted among the Middle Eastern population showed that 36.8% and 26.4% of the participants answered “No” and “Not sure” when asked if they would take the vaccine once it becomes available.^23^ Furthermore, a study presents the largest online survey on vaccine hesitancy that covered a heterogeneous population of Arabic people living all over the globe. From all the 23 Arab countries and territories, the analysis showed a significant rate of vaccine hesitancy among Arabs in and outside the Arab region (83% and 81%, respectively). Additionally, the study revealed the most cited reasons for hesitancy which were concerns about side effects and distrust in healthcare policies, vaccine expedited production, published studies and vaccine producing companies, another factors were related to the age, sex, educational level, and suffering from chronic disease. On top of that, respondents from the Arab Gulf countries (Qatar, Oman, Kuwait, Bahrain, Saudi Arabia, and UAE) plus Libya and Sudan showed the highest willingness for vaccination, while those who showed the least willingness are participants from the west region (Algeria, Tunisia, Mauritania, and Morocco).^24^

In Saudi Arabia, the Ministry of Health launched a vaccine campaign on the December 17, 2020, through a mobile application named Sehaty, which facilitates registration for COVID‐19 vaccination, offering the Pfizer‐BioNTech COVID‐19 Vaccine, and aims to provide free vaccination to all citizens and residents.^25^ Several studies have been conducted to assess the COVID‐19 vaccine hesitancy, in Saudi Arabia, a single study was carried out before the vaccines were approved or launched at a national level, the study showed that 67% of respondents intended to receive the hypothetical vaccine, and only (7%) were hesitant to take it.^26^ Many studies were conducted after administration of the vaccine in Saudi Arabia, where the vaccine acceptance rates range between 31.8% and 71.9%.^26^, ^27^, ^28^, ^29^, ^30^, ^31^, ^32^, ^33^ Saudi Arabia commenced a booster vaccination program in December 2021 for individuals aged 16 years and older, and as of February 14, 2022, only 67.8% of the Saudi population were fully vaccinated.^34^ The risk factors that predict COVID‐19 vaccine hesitancy in Saudi Arabia include sociodemographic factors, such as being married and higher educational level, age, gender, in addition to belief in conspiracy theories and psychosocial factors,^27^, ^28^ a small portion of participants were refusing to receive the vaccine due to the potential long‐term side effects and expedited vaccine trials.^29^

In Jordan, on December 23, 2020 the Ministry of Health in Jordan has launched the national vaccination campaign and invited everyone who lives in Jordan to register for free vaccination against COVID‐19. Around 52.9%–65% of participants suffered before vaccination from vaccine hesitancy.^5^ Three types of vaccines are currently being administered in Jordan: AstraZeneca, Vaxzevria, Pfizer Bionteck (PB), and Sinopharm (SP) vaccines.^3^ Several studies have been conducted in Jordan after administration of the COVID‐19 vaccines; where the vaccine acceptance rates were fairly low (28.4%, 37.4%), where the most trusted sources of information on COVID‐19 vaccines were healthcare providers.^32^, ^35^ In Jordan, higher monthly income was associated with a higher acceptance rate for COVID‐19 and flu vaccines.^32^ Though, a study showed that most of the public trusted that scientists have adequate tools and knowledge to develop a COVID‐19 vaccine and that the development of such a vaccine would put an end to the current pandemic.^36^

When discussing the matter of booster doses; a study discussed the vaccine booster dose acceptance rates in low, middle, and high‐income countries of the East Mediterranean Region showed that the acceptance rates were 73.4%, 67.9%, and 83.0%, respectively. The leading causes behind booster dose rejection were the beliefs that booster doses have no benefit and have severe side effects.^37^ In Jordan, many studies were conducted to assess the acceptance of booster dose, where the acceptance rates range between 44.6% and 72.3%, there were differences in the acceptability of a booster dose for COVID‐19 according to the demographic and clinical characteristics of the participants.^38^, ^39^, ^40^

It was observed that vaccine hesitancy was high earlier and decreased over time with the hope of vaccine efficacy. People in different countries had varying percentages of vaccine uptake (28%–86.1%), vaccine hesitancy (10%–57.8%), vaccine refusal (0%–24%). The most common determinants affecting vaccination intention include vaccine efficacy, vaccine side effects, mistrust in healthcare, religious beliefs, and trust in information sources. Additionally, vaccination intentions are influenced by demographic factors such as age, gender, education, and region.^41^

COVID‐19 doses shot initiatives will almost certainly suffer many difficulties, of which public hesitancy and conspiracy theories towards the vaccine are of great importance.^32^ Hence, it is critical to quantify baseline acceptability levels for the booster dose to create interventions to increase booster dose uptake. As a result, this study aims to assess the acceptability of the COVID‐19 vaccine and the factors that impact the intention to take the COVID‐19 vaccine and its booster dose.

## 2 MATERIALS AND METHODS

### 2.1 Design and setting

A cross‐sectional survey was utilized in this study to assess the acceptability of the COVID‐19 vaccine. This study was conducted in two Arabic countries which are Saudi Arabia and Jordan. A self‐administered web‐based questionnaire was used for data collection created using Survey Monkey, and distributed via social media platforms. Based on the Cochran formula for the large population, the planned sample size was at least 385 participants. Ethical approval and institutional permission were obtained before data collection. Consent was obtained from the participants to participate voluntarily in the study. As well, they will have the right to choose not to participate or withdraw from the research survey at any time. Participants' responses were confidential. The total number of participants who filled out the questionnaire was 518.

### 2.2 Data collection

A self‐administered web‐based questionnaire distributed online was used for data collection. It was adopted from previous studies that assessed COVID‐19 vaccine and its booster dose hesitancy, attitudes toward all vaccines and confidence trends toward the COVID‐19 vaccine.^10^, ^42^, ^43^, ^44^

The questionnaire covers main sections in addition to demographic information (age, gender, region or country, marital status, education); other sections include vaccination status, the intention to have children vaccinated, health insurance, friends or family who tested positive for COVID‐19, living with a vulnerable or immunocompromised person, and pre‐existing conditions. The attitude section had nine items regarding attitudes toward vaccines in general. The last section had 18 items regarding confidence trends with the COVID‐19 vaccine.

### 2.3 Statistical analysis

The Statistical Package for Social Sciences (IBM‐SPSS) was used to analyze the data. Categorical variables were reported as frequency counts and percentages. Whereas continuous variables were reported as mean and standard deviation. Moreover, a cross‐tabulation analysis using the *χ* ^2^ test was conducted to examine significant differences between categorical variables. In addition, logistic regression was used to compute the adjusted odds ratio (95% CI) and to investigate the relationship between a Binary response variables and a set of explanatory, or independent, variables. *T* test and ANOVA were used to examine significant differences between continuous variables.

## 3 RESULTS

In this study, among 518 participants, 72.4% are female and 27.6% are male. The response rate is higher in Jordan than in Saudi Arabia (76.1% vs. 23.9%). Most of the respondents have a bachelor's degree (58.2%). The majority of participants are in two age groups: 25–34 and 35–44 years' groups (28.4% and 31.3%, respectively). The majority of the participants (63.5%) are married, and 60.4% of them have children. Of the participants, 70.8% are working in the medical field, and 14.9% have chronic diseases.

The sociodemographic and professional characteristics of each country are shown in Table 1. The majority are females in both countries. Most of the participants have a bachelor's degree, are married with children working in the medical field, and don't have chronic diseases in both countries. Most of the Jordanian participants are in the 25–34 age group, while the Saudi participants are in the 35–44 age group.

**Table 1:** Sociodemographic and professional characteristics according to the country.

Characteristics	Category	Total *N* = 518		Jordan *N* = 394		Saudi Arabia *N* = 124	
		*N*	%	*N*	%	*N*	%
Gender	Male	143	27.6	111	28.2	32	25.8
	Female	375	72.4	283	71.8	92	74.2
	Total	518		394		124	
Education level	Less than high school	25	4.8	14	3.6	11	8.9
	Diploma	42	8.1	35	8.9	7	5.6
	Bachelor	301	58.2	228	58	73	58.9
	Higher education	149	28.8	116	29.5	33	26.6
	Total	517		393		124	
Marital status grouped	Married	329	63.5	242	61.4	87	70.2
	Not married	189	36.5	152	38.6	37	29.8
	Total	518		394		124	
Age group	18–24	100	19.3	77	19.5	23	18.5
	25–34	147	28.4	118	29.9	29	23.4
	35–44	162	31.3	117	29.7	45	36.3
	45–54	79	15.3	59	15	20	16.1
	Above 55	30	5.8	23	5.8	7	5.6
	Total	518		394		124	
Having children	Yes	313	60.4	226	57.4	87	70.2
	No	205	39.6	168	42.6	37	29.8
	Total	518		394			
Working in the medical field	Yes	367	70.8	336	85.3	31	25
	No	151	29.2	58	14.7	93	75
	Total	518	100.0	394		124	
Having chronic diseases?	Yes	77	14.9	55	14	22	17.7
	No	441	85.1	339	86	102	82.3
	Total	518	100.0	394		124	

Table 2 provides insights into respondents' perceptions and practices related to COVID‐19 infection and the COVID‐19 vaccine, categorized by country (Jordan and Saudi Arabia). Regarding COVID‐19 vaccination, most of the participants received two doses of the COVID‐19 vaccine (54.4%), a higher percentage of respondents in Jordan received two doses of the vaccine compared to Saudi Arabia. However, Saudi Arabia had a higher proportion of respondents who received a booster dose. Nearly, 42% of the participants had already taken a booster dose which declared good acceptance. In terms of intentions for a booster dose, out of the participants who didn't receive the booster dose, 19.9% declared a definite willingness to receive it, a higher percentage of respondents in Saudi Arabia expressed willingness to get a booster compared to Jordan. Regarding the participants who didn't receive any dose of the vaccine, there were 25% who declared an intention to get the vaccine. Most of them were encouraged to get the vaccine via laws and instructions issued by the government, followed by reliance on research results and scientific references. The usefulness of applications for following up on the vaccine was generally perceived positively by respondents in both countries, with a higher percentage of respondents in Saudi Arabia reporting the applications to be helpful.

**Table 2:** Respondents' perceptions and practices related to the COVID‐19 infection and COVID‐19 vaccine according to the country.

Question	Category	Total *N* = 518		Jordan *N* = 394		Saudi Arabia *N* = 124		*p* Value
		*N*	%	*N*	%	N	%	
Did you receive the COVID‐19 vaccine?	Yes, one dose	10	1.9	8	2	2	1.6	<.001*
	Yes, two doses	282	54.4	257	65.2	25	20.2	
	Yes, booster	218	42.1	122	31	96	77.4	
	No	8	1.5	7	1.8	1	0.8	
	Total	518		394		124		
Are you going to get the booster dose?	Yes	58	19.9	44	16.7	14	50	<.001*
	No	233	80.1	219	83.3	14	50	
	Total	291		263		28		
Did the applications help you to follow up on your vaccine?	Yes	441	87.0	321	83.4	120	98.4	<.001*
	No	66	13.0	64	16.6	2	1.6	
	Total	507		385		122		
Were you infected with COVID‐19?	Yes, once	255	49.4	204	51.9	51	41.5	<.001*
	Yes, more than one time	87	16.9	81	20.6	6	4.9	
	No	174	33.7	108	27.5	66	53.7	
	Total	516		393		123		
How do you describe the symptoms?	Mild	75	22.1	65	23	10	17.9	.591
	Moderate	176	51.9	147	51.9	29	51.8	
	Severe	88	26.0	71	25.1	17	30.4	
	Total	339		283		56		
Did you lose any of your family members because of COVID‐19?	Yes	142	28.2	115	30.1	27	22.3	.105
	No	361	71.8	267	69.9	94	77.7	
	Total	503		382		121		
Do you agree with Children's COVID‐19 vaccination?	Yes	135	26.8	98	25.7	37	30.6	.287
	No	368	73.2	284	74.3	84	69.4	
	Total	503		382		121		
Do you Live with a vulnerable/immunocompromised person?	Yes	98	21.3	78	22	20	18.9	.589
	No	363	78.7	277	78	86	81.1	
	Total	461		355		106		

* *p* Value is significant at the .05 level or less.

A significant proportion of respondents in both countries reported being infected with COVID‐19, 49.4% of the participants got infected once, 16.9% got infected more than once, 51.9% had moderate, and the majority of them (42.3%) got infected before the vaccine symptoms, while 33.7% have never gotten infected, with the frequency of infection being slightly higher in Jordan. Symptom severity did not differ remarkably between the two countries, with respondents reporting mild, moderate, and severe symptoms in similar proportions.

The loss of family members due to COVID‐19 was reported by a substantial number of respondents in both countries in addition to living with vulnerable or immunocompromised individuals (71.8% and 78.7%, respectively)., with no significant difference between Jordan and Saudi Arabia. Opinions on children's COVID‐19 vaccination were mixed, with a majority of respondents in both countries expressing agreement (73.2%), but no significant difference was observed between the two countries.

In summary, the significant results from Table 2 indicate differences between Jordan and Saudi Arabia in terms of COVID‐19 vaccination rates, intentions for a booster dose, perception of application usefulness, and COVID‐19 infection rates (*p* < .001). These findings highlight variations in the perceptions and practices related to COVID‐19 between the two countries.

Table 3 shows the participants' attitudes toward the COVID‐19 vaccine, the majority of participants agreed that COVID‐19 is a dangerous disease (53.6%) and by taking the booster dose, they are protecting people close to them (43.9%). Participants agreed that not only the healthcare workers should get the booster dose (49.2%), It is better to take the vaccine than to get the infection (62.4%), they didn't think that COVID‐19 vaccine is a conspiracy to inflict spy chips in our bodies (67%) or could cause infertility (52.8%), or it is useless because of the many mutations of the virus (40.7%), and they think that the side effects of the vaccine are less important than the risk of the disease itself (48.6%).

**Table 3:** Attitudes toward COVID‐19 vaccines.

Items	Agree		Neutral		Disagree		Total
	*N*	%	*N*	%	*N*	%	
COVID‐19 is a dangerous disease	247	53.6	142	30.8	72	15.6	461
By taking the booster dose, I am protecting the people close to me from COVID‐19	200	43.9	167	36.6	89	19.5	456
It is better to get the infection than to take the vaccine	80	17.4	93	20.2	287	62.4	460
The booster dose can cause symptoms similar to the disease itself	245	53.3	134	29.1	81	17.6	460
I will take only two doses of the vaccine because it is mandatory	228	49.9	97	21.2	132	28.9	457
The COVID‐19 vaccine is a conspiracy to inflict spy chips in our bodies	37	8	115	24.9	309	67	461
COVID‐19 vaccine could cause infertility	32	7	184	40.2	242	52.8	458
The side effects of the vaccine are less important than the risk of the disease itself	220	48.6	160	35.3	73	16.1	453
The COVID‐19 vaccine useless because of the many mutations of the virus	118	25.8	153	33.5	186	40.7	457
I will take the booster dose only if it is mandatory	249	54.6	93	20.4	114	25	456
Online applications facilitate the procedures for receiving the COVID‐19 vaccine	358	78.7	75	16.5	22	4.8	455
Online applications motivate people to take the booster dose of the COVID‐19 vaccine	252	55.3	139	30.5	65	14.3	456

On the other hand, there was a negative attitude toward the booster dose. Participants thought that the booster dose could cause symptoms similar to the disease itself (53.3%), they would take only two doses of the vaccine because it was mandatory (49.9%), and they would take the booster dose only if it was mandatory (54.6%).

Regarding the perceptions of online applications; the vast majority agreed that online applications facilitate the procedures for receiving the COVID‐19 vaccine and motivate people to take the booster dose of the COVID‐19 vaccine (78.7% and 55.3%, respectively).

In summary, Table 3 provides insights into participants' attitudes toward COVID‐19 vaccines. While there is general agreement about the dangerous nature of COVID‐19 and the importance of the booster dose, there are differing opinions on topics such as vaccine side effects, vaccine efficacy, and the necessity of the booster dose. Additionally, online applications are perceived positively in terms of facilitating vaccination procedures and boosting motivation for the booster dose.

Table 4 shows the participants' attitudes toward vaccines in general. The vast majority of the participants had a positive attitude toward vaccines in general; most of them agreed that vaccines are effective, important to health, important to the community, and could protect against diseases (67%, 60.9%, 70.5%, and 67.4%, respectively). In addition, they agreed that all vaccines provided by the government program in my community are beneficial, and the information that I receive about vaccines from the vaccine program is reliable and trustworthy (60.5% and 59.8%, respectively). Furthermore, most of them agreed to commit to the National Vaccination Program and follow the doctor's instructions regarding vaccinations (81.9% and 80.1%, respectively).

**Table 4:** Attitudes toward vaccines in general.

Items	Agree		Neutral		Disagree		Total
	*N*	%	*N*	%	*N*	%	
Vaccines are important to health	308	67	110	23.9	42	9.1	460
Vaccines are effective	279	60.9	134	29.3	45	6.5	458
Vaccines are important to the community	325	70.5	106	23	30	6.5	461
All vaccines provided by the government program in my community are beneficial	277	60.5	139	30.3	42	9.2	458
I commit to the National Vaccination Program	376	81.9	68	14.8	15	3.3	459
The information that I receive about vaccines from the vaccine program is reliable and trustworthy	275	59.8	146	31.7	39	8.5	460
Vaccines could protect against diseases	310	67.4	119	25.9	31	6.7	460
I follow the doctor's instructions regarding vaccinations	367	80.1	75	16.4	16	3.5	458

In summary, Table 4 highlights participants' attitudes toward vaccines in general. While there is a general agreement about the importance, effectiveness, and community benefits of vaccines, there are varying levels of trust in government‐provided vaccines and the information provided by vaccine programs. The majority of participants expressed commitment to the National Vaccination Program and reported following doctor's instructions regarding vaccinations.

Attitude scores based on the attitude toward vaccination questions were recognized as attitude questions, and the total score was calculated by computing the scores for these questions. The means are shown below based on different demographic characteristics. Table 5 compares participants' attitude scores based on different factors. It shows that participants' attitudes were significantly associated with educational level (*p* ≤ .001). In addition, the participants' attitudes were significantly associated with age groups (*p* = .032). Participants with higher education levels and older age groups tend to have better attitude toward vaccines.

**Table 5:** Comparison of participants' attitude scores.

Factor	Category	*N*	Mean	SD	*p* Value
Gender	Male	126	23.10	4.06	.373
	Female	324	22.21	3.96	
Country	Jordan	344	22.54	4.066	.442
	Saudi Arabia	106	22.20	3.80	
Educational level	Less than high school	20	20.55	5.28	<.001*
	Diploma	36	20.94	4.03	
	Bachelor	257	22.29	4.00	
	Higher education	136	23.45	3.56	
Marital status	Married	286	22.78	4.06	.713
	Not married	164	21.90	3.85	
Age groups	18–24	80	21.45	3.70	.032*
	25–34	132	21.96	3.87	
	35–44	138	22.83	4.08	
	45–54	72	23.03	4.28	
	Above 55	28	24.39	3.40	
Medical field work‐related	Yes	324	22.69	3.89	.186
	No	126	21.87	4.24	
Do you have a chronic disease	Yes	69	23.49	3.39	.354
	No	381	22.27	4.08	

* *p* Value is significant at the .05 level or less.

Table 6 shows the association between demographic data (gender, age, education level, nature of the job, and health status) and the intention to receive a vaccine booster dose. There was a significant association between the intentions to receive a vaccine booster dose in the country, as the Saudi participants are seven times more likely to be willing to get the booster dose compared to Jordanian participants (*p* < .001). Participants who did not encourage the vaccine for children were less likely to get the booster dose (*p* < .001).

**Table 6:** Factors associated with getting the COVID‐19 booster dose.

Factor	Category	Will you get the booster dose				Odds ratio (CI 95%)	*p* Value
		Yes (*N* = 58)		No (*N* = 233)			
		*N*	%	*N*	%		
Gender	Male	14	19.4	58	80.6		1
	Female	44	20.1	175	79.9		
	Total	58	19.9	233	80.1		
Country	Jordan (reference)	44	16.7	219	83.3	7.165	<.001*
	Saudi Arabia	14	50	14	50		
	Total	58	19.9	233	80.1		
Educational level	Less than high school	2	20	8	80		.989
	Diploma	6	22.2	21	77.8		
	Bachelor	34	19.4	141	80.6		
	Higher education	16	20.3	63	79.7		
	Total	58	19.9	233	80.1		
Marital status	Married	33	19.4	137	80.6		.793
	Not married	25	20.7	96	79.3		
	Total	58	19.9	233	80.1		
Age group	18–24	14	22.6	48	77.4		.676
	25–34	21	21	79	79		
	35–44	13	15.7	70	84.3		
	45–55	7	18.9	30	81.1		
	Above 55	3	33.3	6	66.7		
	Total	58	19.9	233	80.1		
Do you have children	Yes	31	19.1	131	80.9		.703
	No	27	20.9	102	79.1		
	Total	58	19.9	233	80.1		
Health field work‐related	Yes	42	18.7	183	81.3		.319
	No	19	24.2	50	75.8		
	Total	58	19.9	233	80.1		
Do you have a chronic disease	Yes	10	33.3	20	6.7		.059
	No	48	18.4	213	81.6		
	Total	58	19.9	233	80.1		
Applications helped you to get the vaccine	Yes	52	21.3	192	78.8		.179
	No	6	12.8	41	87.2		
	Total	58	19.9	233	80.1		
Previous VOVID infection	Yes, once	31	20	124	80		.274
	Yes, more than one time	8	13.6	51	86.4		
	No	19	24.7	58	75.3		
	Total	58	19.9	233	80.1		
Symptoms	Mild	12	22.6	41	77.4		.580
	Moderate	17	15.9	90	84.1		
	Severe	10	18.5	44	81.5		
	Total	39	18.2	175	81.8		
Family member loss due to COVID‐19	Yes	19	24.4	59	75.6		.267
	No	38	18.4	168	81.6		
	Total	57	20.1	227	79.9		
Encouraging COVID‐19 vaccine for children	Yes (reference)	25	52.1	23	47.9	0.123	<.001*
	No	33	13.9	204	86.1		
	Total	58	20.4	227	79.6		
Living with a vulnerable person	Yes	13	22	46	78		.546
	No	37	18.5	163	81.5		
	Total	50	19.3	209	80.7		

* *p* Value is significant at the .05 level or less.

Table 7 provides insights into the factors associated with receiving the COVID‐19 vaccine and its booster dose. There was a significant association between taking the vaccine booster dose in the country, as well as age group, working in the medical field, previous COVID‐19 infection, and the intention to vaccinate the children.

**Table 7:** Factors associated with receiving the COVID‐19 vaccine and its booster dose.

Factor	Category	Did you receive the COVID‐19 vaccine				Odds ratio (CI 95%)	*p* Value
		Received 1 or 2 vaccine doses (*N* = 292)		Received booster (*N* = 218)			
		*N*	%	*N*	%		
Gender	Male	72	24.7	71	32.6		.812
	Female	220	75.3	147	67.4		
	Total	292		218			
Country	Jordan (reference)	265	90.8	122	56	0.086	<.001*
	Saudi Arabia	27	9.2	96	44		
	Total	292		218			
Educational level	Less than high school	10	3.4	14	6.4		.156
	Diploma	27	9.3	14	6.4		
	Bachelor	175	60.1	120	55		
	Higher education	79	27.1	70	32.1		
	Total	291		218			
Marital status	Married	170	58.2	153	70.2		.382
	Not married	122	41.8	65	29.8		
	Total	292		218			
Age group	18–24 (reference)	63	21.6	35	16.1	1.336	.030*
	25–34	99	33.9	46	21.1		
	35–44	84	28.8	76	34.9		
	45–55	37	12.7	41	18.8		
	Above 55	9	3.1	20	9.2		
	Total	292		218			
Health field work‐related	Yes (reference)	225	77.1	137	62.8	0.502	.031*
	No	67	22.9	81	37.2		
	Total	292		218			
Do you have a chronic disease	Yes	30	10.3	46	21.1		.225
	No	262	89.7	172	78.9		
	Total	292		218			
Applications helped you to get the vaccine	Yes	243	83.8	198	91.2		.902
	No	47	16.2	19	8.8		
	Total	290		217			
Previous COVID‐19 infection	Yes (reference)	214	73.5	122	56.2	1.697	.025*
	No	77	26.5	95	43.8		
	Total	291					
Symptoms	Mild	52	24.3	22	18.5		.473
	Moderate	108	50.5	65	54.6		
	Severe	54	25.2	32	26.9		
	Total	214		119			
Family member loss due to COVID‐19	Yes	78	27.8	62	29.5		.615
	No	207	72.6	148	70.5		
	Total	285		210			
Children COVID‐19 vaccine	Yes (reference)	48	16.8	86	41.1	3.879	<.001*
	No	238	83.2	123	58.9		
	Total	286		209			
Living with a vulnerable person	Yes	59	22.7	36	18.7		.351
	No	201	77.3	157	81.3		
	Total	260		193			

* *p* Value is significant at the .05 level or less.

## 4 DISCUSSION

To our knowledge, this is one of the first studies to assess the acceptability of the COVID‐19 vaccine and the factors that impact the intention to take the COVID‐19 vaccine and its booster dose among adults in two Arab countries. Factors at the individual level were determinants of acceptance to receive the COVID‐19 vaccine and its booster dose. Our findings provided a piece of knowledge for developing behavioral interventions to increase COVID‐19 vaccine uptake among Jordanian and Saudi adults. During the study period, 54.4% of participants received two doses of the COVID‐19 vaccine, which is comparable to the worldwide rate, where 69.9% of the world's population has received at least one dose of the COVID‐19 vaccine.^45^ However, the prevalence of COVID‐19 vaccine booster dose uptake among our participants (19.9%) was lower than that of older adults in the United States (44%). Given the high effectiveness of the booster dose in preventing severe consequences and deaths associated with COVID‐19.^46^ There is a strong need to increase COVID‐19 vaccine booster dose coverage among adults. Only 38.1% of the participants were hesitant to take the COVID‐19 booster dose, which is comparable to the finding in Greece (38.1%).^47^ In contrast, the study results were much higher than the finding in China (17.2%–18.3%).^48^

Our findings provided some empirical insights for developing interventions to decrease vaccine hesitancy about receiving the COVID‐19 vaccine booster dose, since the intention to receive a vaccine booster dose was significantly lower among Jordanian participants, so more efforts should be implicated in Jordan. In addition, there was a significant association between taking the vaccine booster dose in the country, as well as age group, working in the medical field, and the intention to vaccinate the children. The history of COVID‐19 infection was also a facilitator for receiving the COVID‐19 vaccine booster dose in our sample.

The results of the present study indicated good acceptance of the vaccination with COVID‐19 and its booster dose. This implies the existence of a good attitude toward vaccination and its importance. It is important to address attitudes among participants, as they are significantly associated with higher hesitancy to receive the booster dose. Our results show that participants' positive attitudes were significantly associated with educational level since those with higher education had a more positive attitude toward the vaccines; such a finding was similar to that of a previous study in Hong Kong.^49^ Therefore, health authorities' announcements promotingthe COVID‐19 vaccine booster dose should be straightforward and easy to understand for people with low educational levels. In addition, the participants' attitudes were significantly associated with age groups; those in the 45–54 age group had a more positive attitude toward the vaccines.

According to 87% of participants, the use of applications helped them follow their vaccines. Following consideration of the COVID‐19 pandemic by the WHO, governments have established applications to communicate with citizens regarding vaccinations and other matters.^2^, ^3^ It is essential to emphasize that smartphone applications, for example, were shown to be effective.^50^

Approximately half of the participants were infected with COVID‐19 once, and slightly more than a quarter were infected more than once. However, statistics from the United States showed that 60% of people were infected with COVID‐19. The reason for this can be attributed to the early efforts placed by the government compared with those in the United States.^51^

This study highlights that participants expressed positive attitudes toward receiving a vaccination; this was better than that reported in another study.^44^ We think that this is plausible due to the daily cases of high mortality rates resulting from COVID‐19. On the other hand, negative attitudes toward the booster dose were shown by participants due to their thoughts about having possible related symptoms like the disease itself. It is possible to explain this result by negative or wrong information resulting from the media. Many people questioned vaccinations from different perspectives, which confused the public.^52^, ^53^

The results showed that the Saudi participants are seven times more likely to be willing to get the COVID‐19 vaccine compared to the Jordanian participants. What was of greater interest was the attitude of participants from Saudi Arabia toward having a booster dose compared with those from Jordan. This could be explained by the fact that a majority of people who did not receive vaccination suffered as a result, making them more interested in receiving a booster dose of vaccination. This trend is more strongly supported by the responses to the second question, “Are you going to get the booster dose.” In this context, half of the participants from Saudi Arabia had the intention to take the booster dose, compared to 17% of participants from Jordan.

Participants who do not encourage the COVID‐19 vaccine for children are less likely to be willing to get the booster dose. It seems to be a logical outcome given that those people may believe that the vaccine is harmful to children and could get the first and second doses as mandatory. Moreover, the use of applications seems to be more effective among participants from Saudi Arabia compared with their counterparts from Jordan. This may be due to the fact that the area of Saudi Arabia is larger than that of Jordan, a matter that makes the application more effective. Therefore, a potential future necessity to consider the planning of messaging about receiving the COVID‐19 booster dose vaccine may require some communication efforts.

## 5 STUDY LIMITATIONS

Vaccine acceptance and hesitancy change over time based on evolving evidence and vaccination data, necessitating longitudinal studies to understand individuals' attitudes and preference shifts regarding COVID‐19 vaccination over time. The over‐representation of Jordan and Saudi Arabia introduces biased findings, limiting generalizability to other Arab continents, highlighting the urgency for studies on different continents. Another limitation of our study is that it arises from a cross‐sectional design where the data reflects a snapshot of willingness to take the COVID‐19 vaccine when, in reality, individual attitudes are dynamic and evolving. Finally, COVID‐19 vaccine uptake should be investigated in the future since intentions do not always predict individuals' actions.

## 6 CONCLUSION

In conclusion, the findings of this study provide valuable insights into the sociodemographic characteristics, perceptions, and practices related to COVID‐19 infection and vaccination among participants in Jordan and Saudi Arabia.

The findings revealed notable distinctions between the two countries, with Jordan demonstrating higher overall COVID‐19 vaccination rates (two doses), while Saudi Arabia exhibited a greater proportion of participants receiving COVID‐19 booster dose. Meanwhile, COVID‐19 vaccination was generally well accepted, with approximately one‐fifth of the vaccinated participants expressing a definite willingness to receive a booster dose. In particular, Saudi participants demonstrated a significantly higher willingness to receive the booster dose compared to Jordanians. Several factors were found to be associated with the likelihood of receiving the booster dose, including country of residence, age group, occupation in the medical field, previous COVID‐19 infection, and intention to vaccinate children. Additionally, educational level and age group were identified as factors influencing participants' attitudes toward COVID‐19 vaccination. Notably, participants generally held positive perceptions toward vaccine utilization, with Saudi participants expressing a higher degree of perceived usefulness. Additionally, the study emphasizes the importance of online applications in facilitating vaccination procedures and promoting vaccine uptake, with Saudi participants finding them more helpful.

These findings provide important insights for policymakers and healthcare professionals in formulating targeted strategies and interventions to address the challenges and promote vaccination uptake. It also highlights the need for tailored communication and educational campaigns that address specific concerns and misconceptions regarding vaccine side effects, efficacy, and the necessity of booster doses. Intervention strategies aimed at addressing knowledge gaps, improving attitudes, and enhancing the intention to receive the COVID‐19 vaccine should prioritize individuals with lower educational levels, those older than 55 years, those not working in the healthcare field, and the Jordanian population as a whole. Further research and continuous monitoring are essential to better understand and address the evolving perceptions and practices surrounding COVID‐19 vaccination in different populations.

## AUTHOR CONTRIBUTIONS

Sawsan Mubarak, Ashraf A'aqoulah, Samir Albalas, Hadeel AlGhawrie, and Nisreen Innab performed the research. Sawsan Mubarak, Ashraf A'aqoulah, and Hadeel AlGhawrie designed the research study. Ashraf A'aqoulah, Nisreen Innab, and Samir Albalas wrote the methods. Sawsan Mubarak and Hadeel AlGhawrie analyzed the data. Samir Albalas and Ashraf A'aqoulah wrote a discussion. Sawsan Mubarak, Ashraf A'aqoulah, Samir Albalas, Hadeel AlGhawrie, and Nisreen Innab reviewed and edited the manuscript. All authors have read and approved the final manuscript.

## CONFLICT OF INTEREST STATEMENT

The authors declare no conflict of interest.

## ETHICS STATEMENT

This study was approved by the Institutional Review Board (IRB) at King Abdullah International Medical Research Centre (KAIMRC). This study approval number is NRC22R/275/06.

## Data Availability

The data that support the findings of this study are available on request from the corresponding author.
